# Follow the money: how is medical school teaching funded?

**DOI:** 10.1192/bjb.2020.50

**Published:** 2021-04

**Authors:** Aileen O'Brien, Ania Korszun

**Affiliations:** 1 St. George's University of London, UK; 2 Barts and The London School of Medicine and Dentistry, UK

**Keywords:** Education and training, recruitment, educational policy, medical schools, undergraduate teaching

## Abstract

Growing student numbers are producing greater demand for teaching, and resources allocated for education are being placed under increasing strain. The need for more student clinical placements and more clinician teaching time is expanding. Psychiatrists have successfully drawn attention to the importance of parity between mental and physical illness. We now have a responsibility to ensure enhanced opportunities to teach psychiatry to our medical students. This is set against a background of an increasing number of psychiatry consultants leaving the profession and an already stretched National Health Service environment. Many consultants contribute to teaching but do not have this activity included in their job plans. Although clinics and clinical meetings are inevitably slower when students are present, there is often no backfill provided. As outlined below, trusts receive substantial funding to cover costs related to the teaching of medical students, but most of us don't know what actually happens to this money. Here, we discuss how teaching is currently funded and make recommendations regarding improving accountability.

As increasing student numbers cause greater demand for teaching, clinicians may struggle to meet the conflicting pressures on their time. This is against a background of an increasing number of psychiatry consultants leaving the profession and an already stretched National Health Service (NHS) environment.

Many consultants who contribute to teaching do not have this activity included in their job plans. Clinics and clinical meetings are inevitably slower when students are present, but there is often no backfill provided. The psychiatry recruitment crisis has been successfully challenged by the Choose Psychiatry campaign;^1^ however, to sustain this and encourage retention, jobs need to be manageable and stimulating with contributions to education adequately recognised.

Trusts receive substantial funding to cover teaching medical students, but most of us don't know what actually happens to this money.

In the 1970's film *All the President's Men*, ‘follow the money’ was the catchphrase, suggesting that the way to shine a light on questionable dealing is to follow financial transfers. Although we are not suggesting that the allocation of funding for medical student education is as questionable, systems for funding education are mostly opaque and surprisingly poorly understood.

So, for those who would like to increase their understanding, we offer some answers to the most frequently asked questions.

## Who funds undergraduate teaching?

The world of medical education is nothing if not acronym heavy (Box 1). Health Education England (HEE) was established in the *Health and Social Care Act 2012* as a special health authority within the Department of Health. It is a non-departmental public body which supports the delivery of education, training and development of the NHS health and public health workforce.^2^ It provides oversight of strategic planning and development of the health and public health workforce, and allocates (and accounts for) funding for education and training resources on behalf of the Department of Health and Social Care (DHSC).

**Box 1:** Educational Bodies

HEE	Higher Education England	An executive body of the Department of Health. It provides coordination for education and training within the health and public health workforce in England.
LETBE	Local Education and Training Board	Statutory regional committees of HEE, responsible for workforce planning, and education and training.
OFS	Office for Students	Regulator established in January 2018. It merged HEFCE and OFFA, and inherited their responsibilities, also taking charge of the granting of degree-awarding powers and university titles.
SIFT (now undergraduate medical tariff)	Service Increment for Teaching	NHS levy that was given out as extra funds to NHS institutions that participate in training undergraduate students
OFFA	Office for Fair Access	Safeguarded and promoted fair access to higher education by approving and monitoring access agreements
HEFCE	Higher Education Funding Council for England	Distributed public money for teaching and research to universities and colleges.

From April 2018, the Office for Students (OFS) became the regulatory body for higher education in the UK, bringing together the functions of the Higher Education Funding Council for England (HEFCE), the Office for Fair Access, the Department for Education and the Privy Council in a single organisation.

Undergraduate medical training is essentially funded in England and Wales by:

• DHSC (via HEE) – for clinical placement costs, more for the final years;
• OFS – this is because medicine counts as a ‘high cost’ course as opposed to, for example, many arts courses;^3^
• tuition fees paid by the students.

The OFS gives each university a grant based on the number of medical students at their institution. The numbers of students that universities can admit are regulated by the OFS and controlled through its intake targets.

## How are placements funded?

The money from HEE is now known as the undergraduate medical tariff or ‘the tariff’ (formerly and often still in fact known by professionals as SIFT (Service Increment for Teaching)). The tariff, set by the Secretary of State for Health, is not a direct payment for teaching but is supposed to cover the additional costs incurred by trusts and other placement providers in delivering medical student teaching.

The money is paid directly to the trusts by HEE, and the amount is based on student numbers that are provided by the university (but not on quality of teaching). So, although the universities are responsible for monitoring the quality of their students’ clinical placements, they have little influence on how the tariff funds are disbursed at trust level. The universities do liaise directly with trusts to determine the number of placements offered and monitor the quality of the teaching provided at those placements. If quality standards are not met by the trusts, the university can in theory withdraw those placements and, consequently, the funding that goes with them. However, for many universities there are no alternative trusts that they can approach. The overall training activity provided by trusts, including that for medical students and doctors in training, is governed by individual learning development agreements between trusts and HEE, which list all education, training and learning activities commissioned by HEE.

Increasingly, patients are cared for in primary care, or by third sector or independent providers. This can lead to practical challenges such as enabling patient contact in patients’ homes. Undergraduate education is rarely considered at the commissioning level, so many providers can decide whether or not to participate in teaching; this adds another level of complexity to the university's obligation to monitor the quality of teaching, as well as the practical difficulties of securing placements.

## What is included in the tariff?

The tariff for undergraduate medical placements was introduced in 2013–2014. Tariffs are adjusted by a market forces factor to compensate for the differences in cost of providing training placements in different parts of the country.

The tariff covers funding for all direct costs involved in delivering education and training, and the list provided by the government is comprehensive:^4^

• direct staff teaching time within a clinical placement;
• teaching and student facilities, including access to library services;
• administration costs;
• infrastructure costs;
• pastoral and supervisory support;
• trainee study leave and time for clinical exams;
• health and well-being (excluding any occupational health assessments);
• course fees and expenses (as required to achieve professional registration);
• student/trainee accommodation costs;
• in-course feedback and assessment;
• formal examining;
• staff training and development relating to their educational role.

## What is the tariff worth (for a year's worth of placements)?

In 2019–2020, a non-medical tariff (for an allied health professional (AHP)) is set at £3720, the medical postgraduate tariff is £11 418 and a medical undergraduate tariff is £33 286.

In summary, in England, teaching one medical student in the clinical years is currently supported by:^5^

• the OFS teaching grant – £1500 per student for the non-clinical years and £10 000 per student during the clinical years (depending on holiday entitlement at different schools, this is about £250 per week for the clinical years);
• placement tariff – healthcare providers receive an average tariff of around £36 000 to provide a year's worth of placements to students in the clinical years;
• tuition fees – £9250 per year for all years.

AHPs are a core part of the NHS people plan,^6^ and traditional ‘medical’ tasks and roles are increasingly being taken on by physician associates, advanced nurse associates, nurse prescribers and other AHPs. Although this is welcomed, with many doctors recognising the necessity in terms of long-term workload reduction, in the immediate term, doctors are increasingly asked to teach and supervise AHPs despite the non-medical tariff being substantially lower.

## Where does all the money go and how is it regulated?

In 2007, the British Medical Association investigated the use of the tariff (or SIFT, as it was then known) using the Freedom of Information Act. Of the 33 trusts contacted, 23 either did not respond, did not know or did not detail how the money was spent. From the ten trusts that did respond, the most frequent response was that funding had historically been incorporated into their baseline budgets, and its use was therefore not recorded separately. As one trust responded (Orwell would be impressed), ‘this income … constitutes part of the totality of the Trust's income base and therefore is embedded within the totality of the Trust's expenditure’. Only seven of the 23 trusts could give any information about consultant teaching time, and this tended to consist of a statement that consultants nominally have one PA per week allocated for teaching.^7^

## That was over 10 years ago. Have things moved on?

There has been some progress in that most medical schools have developed a ‘minimum teaching standards’ document that they use as a shared document between them and trusts. New medical schools have had to start this process from scratch and work with their trusts to provide clear explanations of where and how the money is spent. Tariff funding is still often absorbed into trusts’ finances, but increasingly trusts are being asked to demonstrate where the money is actually going.

## That's England; what about the rest of the UK?

Medical universities in Scotland, Wales and Northern Ireland are funded by the devolved governments in very similar ways to HEFCE, although the fee element of their income varies from no fees (Scotland) to lower fees (Wales) to the same as England (Northern Ireland).

Funding for medical education to the medical universities comes from the higher education budget and then goes to the NHS institutions through an equivalent of the tariff. The scheme in Wales is still named SIFT-W (for Wales); in Scotland, it is the ACT (Additional Costs of Teaching); and the Supplement for Undergraduate Medical and Dental Education (SUMDE) in Northern Ireland.^8^

## Conclusion: use it or lose it!

The level of scrutiny regarding this teaching funding currently only goes in one direction. We believe that trusts need to wake up to the fact that if they are being paid for providing teaching to students, they need to be able to demonstrate this in a transparent way. In *All the President's Men*, the assiduous undercover reporters expose Watergate, leading to the downfall of the president of the USA. Following the money when it comes to trust teaching tariffs is likely to show that the funding is generally poorly accounted for and monitored, rather than deliberately diverted. However, in the current financial climate, trusts are unlikely to continue getting these large amounts of money unless they can demonstrate that funds are going where they are meant to go, and where they say they are going, and that the teaching provided is of at least an adequate standard. In the coming year, HEE will be formally gathering information from trusts on how money is being spent, and many trusts are likely to be unprepared. Effective and clear job planning is probably the most obvious way to demonstrate that the funding is going towards the most expensive and essential resource: clinician time. This needs to be evidenced and protected.

With the current workforce challenges, alternatives to consultant teaching should also be considered, and medical students also need teaching and experience of working in a multidisciplinary team. Clinical teaching fellows are higher trainees funded by the tariff, with protected teaching time in their week (typically around 60%), and anecdotally they make a big difference to student experience. AHPs too can usefully support medical student teaching. The use of simulation training and involvement of expert patients, as well as buying sessions for students to attend specialist clinics in other sectors, may also represent good ways of spending the money available.

There is no doubt that by continuing to ‘follow the money’ we can also improve and track where these precious resources are being used to train safe and competent doctors with the necessary skills to look after our patients’ complex needs in the future.

## Addendum in the time of COVID-19

We originally wrote this piece in the now seemingly distant days before the COVID-19 pandemic. The points we made in the conclusion are now even more relevant. New problems arise as we are forced into a situation where the necessary teaching of basic psychiatry skills must be accomplished without students being able to attend hospitals and clinics to get direct clinical experience in psychiatry. Also, these students will be qualifying as doctors at a time when there will be even greater needs for the skills necessary to deal with the epidemic of mental illness that will follow COVID-19. It is therefore essential to accelerate our efforts in developing alternative ways of teaching, but this is a time when consultants and their teams have even greater pressure on their 'time to teach' as they prioritise new clinical commitments that arise from the COVID-19 pandemic.

The use of digital technologies can no doubt play an increasingly valuable role in teaching. However, distance learning alone will not be adequate; this is a time when the input of all our clinical colleagues remains vital. Students are not currently on clinical placements and their dates of return are uncertain. However, the tariff continues to be paid to Trusts and they should not lose sight of their responsibility for active involvement in creatively addressing educational and training needs. In particular, the mental health risks of their patients will persist and be even greater after COVID-19. These will be further exacerbated if we allow our students to leave medical school with inadequate training in psychiatry.

## About the authors

**Aileen O'Brien** is Dean for Students and Reader in Psychiatry and Education at St George's University of London, UK. **Ania Korszun** is Professor at the Wolfson Institute of Preventive Medicine, Barts and the London School of Medicine and Dentistry, UK.
